# *In situ* synthesis of P3HT-capped CdSe superstructures and their application in solar cells

**DOI:** 10.1186/1556-276X-8-106

**Published:** 2013-02-26

**Authors:** Yanling Peng, Guosheng Song, Xianghua Hu, Guanjie He, Zhigang Chen, Xiaofeng Xu, Junqing Hu

**Affiliations:** 1 State Key Laboratory for Modification of Chemical Fibers and Polymer Materials, College of Materials Science and Engineering, Donghua University, Shanghai, 201620, China; 2 Department of Applied Physics, Donghua University, Shanghai, 201620, China

**Keywords:** P3HT-capped CdSe superstructures, Photoabsorption, Continuous interpenetrating networks, Solar cell

## Abstract

Organic/inorganic hybrid solar cells have great potentials to revolutionize solar cells, but their use has been limited by inefficient electron/hole transfer due to the presence of long aliphatic ligands and unsatisfying continuous interpenetrating networks. To solve this problem, herein, we have developed a one-pot route for *in situ* synthesis of poly(3-hexylthiophene) (P3HT)-capped CdSe superstructures, in which P3HT acts directly as the ligands. These CdSe superstructures are in fact constructed from numerous CdSe nanoparticles. The presence of P3HT ligands has no obvious adverse effects on the morphologies and phases of CdSe superstructures. Importantly, higher content of P3HT ligands results in stronger photoabsorption and fluorescent intensity of CdSe superstructure samples. Subsequently, P3HT-capped CdSe superstructures prepared with 50 mg P3HT were used as a model material to fabricate the solar cell with a structure of PEDOT:PSS/P3HT-capped CdSe superstructures: P3HT/Al. This cell gives a power conversion efficiency of 1.32%.

## Background

The quest and demand for clean and economical energy sources have increased the interest in the development of solar applications. In particular, direct conversion of solar energy to electrical energy using photovoltaic cells has attracted much attention for several decades [1-4]. Among various photovoltaic cells, organic polymer-based solar cells have received considerable attention as a new alternative photovoltaic technology due to their flexibility, light weight, low-cost fabrication, and easy integration into a wide variety of devices [5]. Importantly, bulk heterojunction (BHJ) solar cells based on intimate blends of organic polymer as the donor and inorganic nanomaterials as the acceptor are currently attracting increasingly widespread scientific and technological interests because of the advantages, resulting from these two types of materials, such as low cost, outstanding chemical and physical properties, easy preparation from organic polymers, high electron mobility, excellent chemical and physical stabilities, size tunability, and complementary light absorption from inorganic semiconductors [6-8]. Various organic–inorganic hybrid solar cells have been reported based on the conjunction of organic polymers, such as poly(3-hexylthiophene) (P3HT) [9-12], poly(3,4-ethylenedioxythio-phene) with poly-(styrene sulfonate) (PEDOT:PSS) [13], poly[2-methoxy-5-(3^′^,7^′^-dimethyloctyloxy)-1,4-phenylenevinylene] (MDMO-PPV) [14], and poly(2-methoxy,5-(2-ethyl-hexyloxy)-*p*-phenyl vinylene) (MEH-PPV) [15,16], and inorganic nanocrystals, such as CdSe nanorods [17], hyperbranched CdSe nanocrystals [9,14,18], ZnO [19,20], PbS [10,21], Sb_2_S_3_[11,12], and Si nanocrystals [22].

In these organic–inorganic hybrid solar cells, the polymer as the donor can be excited by solar light, resulting in the generation of strong-bound excitons that can be dissociated at the interface between the polymer and inorganic nanocrystals [23]. Thus, the interface between the polymer and inorganic nanocrystals plays a very important role. Unfortunately, inorganic nanocrystals used as the acceptor are typically capped with organic aliphatic ligands, such as trioctylphosphine oxide (TOPO) [24] and oleic acid (OA) [16]. The presence of organic aliphatic ligands prevents electron transferring from the photoexcited polymer to the nanoparticles [25].

To solve this problem, three strategies have been developed. The first strategy is to prepare inorganic nanocrystals capped with thermally cleavable solubilizing ligands and then heat the nanocrystals for shortening the ligands [26]. However, there are very limited kinds of thermally cleavable solubilizing ligands. The second strategy involves replacing the original long organic layer with short ligands. For example, pyridine [16,24,27], *tert*-butylthiol, [28,29], or acetate acid [9] treatment methods have been used to remove TOPO and OA. However, these processes may be costly and complicated, and precise control of some factors (such as exchange rates) may be difficult. The last strategy is to directly synthesize hybrid inorganic nanocrystals that are capped with donor polymer such as P3HT [30] or PPV [31]. The negative effects of the capping organic aliphatic ligands on charge exchange are eliminated, and the step of transferring inorganic nanocrystals into the polymer solution for exchange can be bypassed, achieving direct synthesis of nanoparticles with photoelectronic polymers as ligands. To this day, several kinds of hybrid inorganic nanocrystals have been well developed for BHJ solar cells, including P3HT-capped CdS single-crystal nanorods [30], MDMO-PPV-capped PbS quantum dots [31], MEH-PPV-capped PbS nanorods [1], and MEH-PPV-capped PbS nanocrystals [32]. It should be noted that these nanoparticles usually have very small diameters (2 to 5 nm), and thus, it is difficult for them to form a well continuous inorganic network, leading to the difficulty of electron transfer and low photoelectric conversion efficiency [33]. Fortunately, it has been found that the shapes of inorganic nanocrystals have a strong effect on the formation of continuous inorganic network in BHJ solar cells [34]. For example, the BHJ solar cells based on CdSe inorganic nanostructures including nanorods [17,35] or nanobranches [36,37] have better continuous interpenetrating networks and thus exhibit more superior photoelectric performances compared with the cells based on CdSe nanoparticles. Furthermore, compared with CdSe nanorods and nanobranches, spherical superstructures constructed by nanosubstructures may be more suitable to form well continuous inorganic network.

To the best of our knowledge, there is no report on the synthesis of inorganic superstructures capped with conductive donor polymer for BHJ solar cells. In this report, we employed P3HT as the ligands to synthesize P3HT-capped CdSe superstructures in a mixed solution of 1,2,4-trichlorobenzene (TCB) and dimethyl sulfoxide (DMSO). This synthetic procedure yielded homogeneous CdSe superstructures that were constructed by 5- to 10-nm CdSe nanoparticles. These P3HT-capped CdSe superstructures can be dissolved in many kinds of solvents, such as 1,2-dichlorobenzene and chloroform, from which thin films can be readily cast to fabricate BHJ solar cells.

## Methods

All of the chemicals were commercially available and were used without further purification. Cadmium acetate dihydrate (Cd(CH_3_COO)_2_·2H_2_O), selenium (Se), DMSO, isopropyl alcohol ((CH_3_)_2_CHOH), ethanol, chloroform (CHCl_3_), TCB, and sodium hydroxide (NaOH) were purchased from Sinopharm Chemical Reagent Co., Ltd. (Shanghai, China). The PEDOT:PSS solution (solvent H_2_O, weight percentage 1.3%) was obtained from Sigma-Aldrich Corporation (St. Louis, MO, USA). The fluorine tin oxide (FTO)-coated glass (resistivity 14 Ω/sq) was purchased from Georgia & Education Equipment Co., Ltd. (Wuhan, China). P3HT was bought from Guanghe Electronic Materials Co., Ltd. (Luoyang, China).

### Synthesis of CdSe superstructures and P3HT-capped CdSe superstructures

In a typical synthesis, Cd(CH_3_COO)_2_·2H_2_O (0.133 g) as precursor was dissolved in the mixture of TCB (16 mL) and DMSO (8 mL) in a three-neck round-bottom flask. After magnetically stirring for 30 min, different amounts (0, 10, 50, or 100 mg) of P3HT were added into the mentioned solutions, and the color of the solution became dark red immediately. The solution was held at 100°C for 30 min with stirring magnetically and purging periodically with dry nitrogen to remove residual water and oxygen, and then the color of the solution became red. Subsequently, this solution was heated to 180°C with the protection of dry nitrogen. In addition, another TCB solution (8 mL) containing Se powder (0.019 g) was heated to 180°C until a transparent red solution was obtained and then injected to the mentioned solution in a three-neck round-bottom flask. After a 10-min reaction at 180°C, the mixture was then cooled to room temperature, isolated via centrifugation at 8,000 rpm, and washed in ethanol three times.

### Fabrication of solar cells

A part of the conductive layer of FTO block was removed by 1 mol/L hydrochloric acid solution containing zinc powder. The FTO-coated glass was ultrasonically cleaned by detergent, saturation (CH_3_)_2_CHOH solution of NaOH, deionized water, and ethanol. The PEDOT:PSS solution was filtered by a 450-nm membrane and spun at the speed of 4,000 rpm to form the PEDOT:PSS layer with a thickness of 120 nm on FTO glass. The PEDOT:PSS layer (about 120-nm thick), as the anode, was annealed at 120°C for 30 min. Subsequently, P3HT-capped CdSe/CdSe sample (20 mg) and P3HT (5 mg) were dispersed in CHCl_3_ solution (1 mL). This solution was filtered by a 450-nm membrane and spun to form about 450-nm-thick CdSe film on PEDOT:PSS layer, and then two drops of CHCl_3_ solution containing 4 mg/mL P3HT were spun on the earlier CdSe layer. Afterwards, this as-fabricated device was annealed at 150°C for 30 min. Finally, an Al layer (about 100-nm thick) was sputtered for 50 min in a metal mask under 4 Pa of argon environment. This Al layer acted as the cathode in the as-fabricated solar cell device. The resulting solar cell device had a structure of FTO/PEDOT:PSS/P3HT-capped CdSe superstructures:P3HT/Al.

### Characterizations

The sizes and morphologies of CdSe superstructures and P3HT-capped CdSe superstructures were investigated by scanning electron microscopy (SEM) (Hitachi S-4800, Hitachi High-Tech, Minato-ku, Tokyo, Japan) and transmission electron microscopy (TEM) (JEM-2010F, JEOL Ltd., Akishima, Tokyo, Japan). The X-ray diffraction (XRD) (Rigaku D/max-g B, Rigaku Corporation, Tokyo, Japan) measurement was carried out using a Cu-K*α* radiation source (*λ* = 1.5418 Å). Fourier transform infrared (FTIR) spectra of ligands in CdSe were obtained by measuring pellets of KBr and sample using an FTIR-Raman spectrometer (Thermo Fisher Scientific, Waltham, MA, USA). A UV–vis spectrophotometer and a fluorescence spectrometer (FP-6600, JASCO Inc., Easton, MD, USA) were used for the optical measurements of CHCl_3_ solution (0.04 mg/mL) containing CdSe superstructures, P3HT-capped CdSe superstructures, and P3HT, respectively. The thermogravimetric analysis (TGA) measurements of the samples were done using the Discovery TGA instrument (TA Instruments, New Castle, DE, USA) under a nitrogen flow rate of 50 mL/min at the heating rate of 10°C/min from 50°C to 600°C.

The photocurrent density-voltage curves of solar cells were measured under illumination (100 mW cm^−2^) using a computerized Keithley model 2400 source meter unit (Keithley Instruments Inc., Cleveland, OH, USA) and a 300-W xenon lamp (69911, Newport Corporation, Irvine, CA, USA) serving as the light source.

## Results and discussion

**Figure 1:**
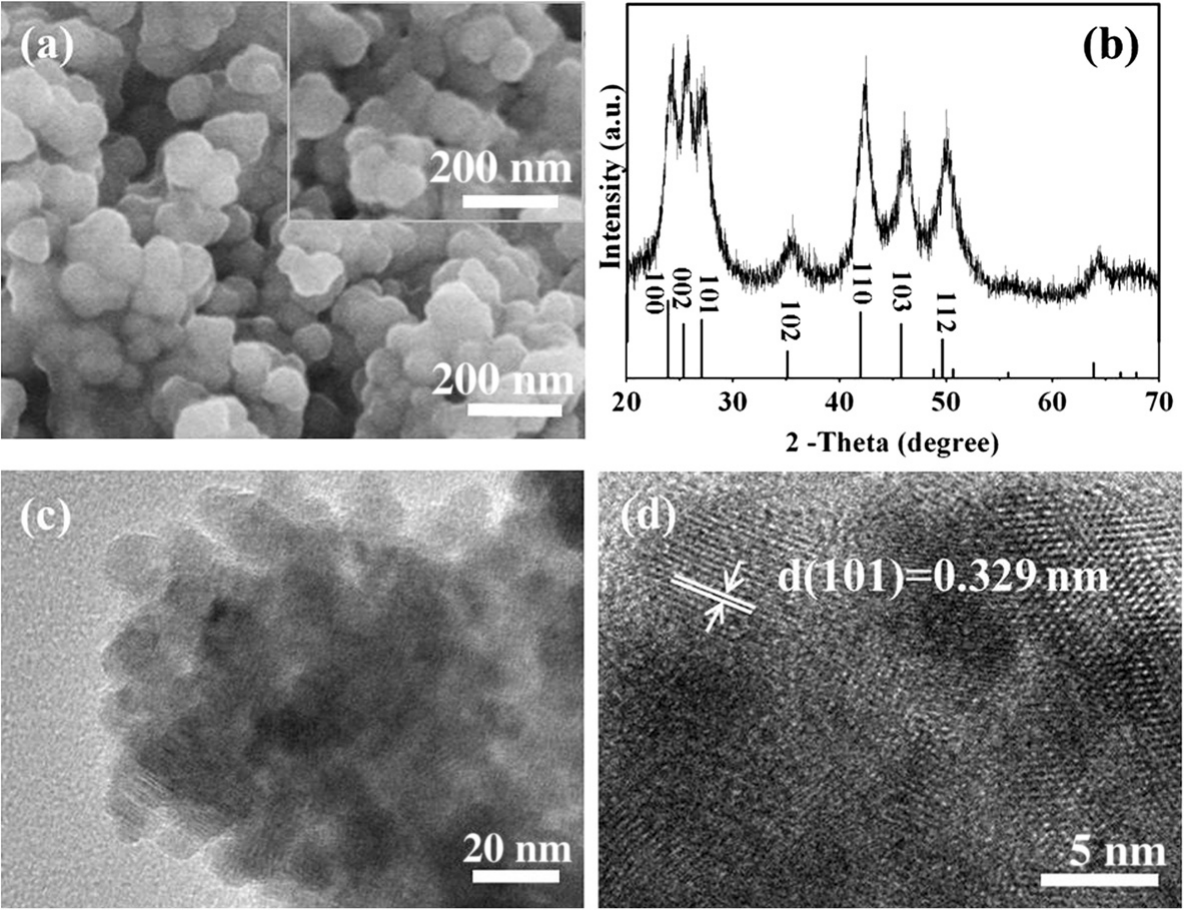
**Overall morphological characterization and XRD analysis of CdSe superstructures. (a)** SEM images of CdSe superstructures (inset: CdSe superstructures synthesized with 50 mg P3HT) and **(b)** XRD pattern of CdSe superstructures. **(c)** TEM and **(d)** HRTEM images of CdSe superstructures synthesized with 50 mg P3HT.

Firstly, the effects of the amount of P3HT on the shapes and phases of CdSe have been investigated. In the absence of P3HT, the CdSe sample has a spherical morphology with a diameter of about 100 nm (Figure 1a). The XRD pattern (Figure 1b) of CdSe superstructures reveals a typical hexagonal wurtzite structure, which is in good agreement with that in literatures [38,39] and from the Joint Committee on Powder Diffraction Standards (JCPDS) (card number 08–0459). These peaks at 23.901°, 25.354°, 27.080°, 35.107°, 41.968°, 45.788°, and 49.669° are assigned to (100), (002), (101), (102), (110), (103), and (112) planes of the CdSe material, respectively. Importantly, this CdSe sample exhibits a pure hexagonal wurtzite structure. When 10, 50, and 100 mg P3HT content were added, the morphology (Figure 1a, inset) and the phase of the CdSe sample were similar to those of the CdSe sample synthesized without P3HT. This indicates that the addition of P3HT has no obvious effects on the shapes and phases of CdSe. To further analyze CdSe superstructures, TEM was used to investigate the model sample prepared using 50 mg P3HT. Interestingly, these CdSe superstructures (Figure 1c) are in fact constructed with numerous CdSe nanoparticles with diameters of 5 to 10 nm. The HRTEM image (Figure 1d) shows well-resolved lattice fringes, demonstrating a high crystalline nature. The *d* spacing of 0.329 nm corresponds to the distance of the (101) planes, which is in agreement with that of the CdSe crystal, by referring to the JCPDS card (number 08–0459).

**Figure 2:**
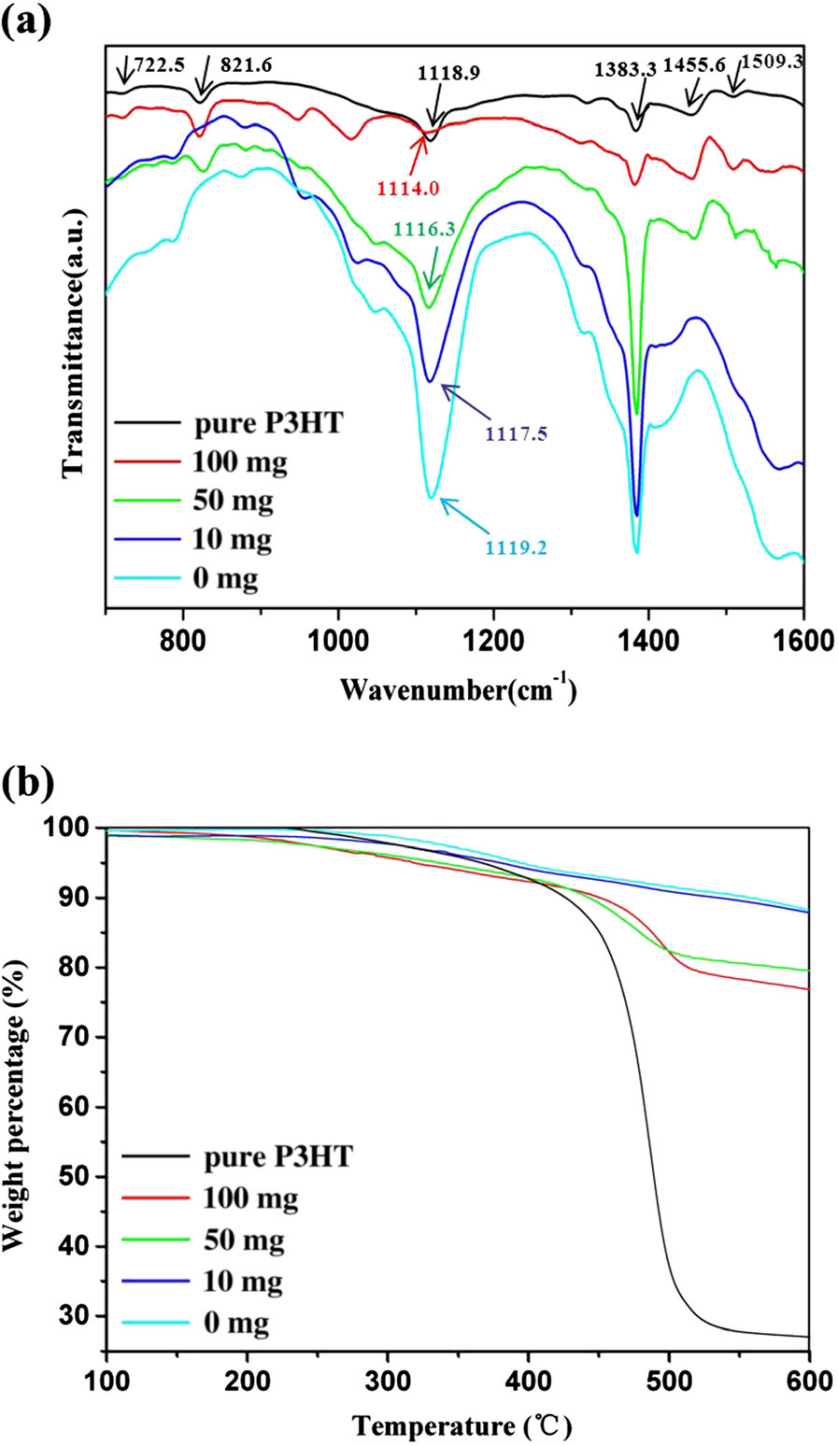
**FTIR spectra and TGA curves. (a)** FTIR spectra and **(b)** TGA curves of pure P3HT and P3HT-capped CdSe superstructures synthesized with different amounts of P3HT at 0, 10, 50, and 100 mg.

Surface ligands of CdSe superstructures are important for their applications in solar cells. The capping ligands of CdSe superstructures prepared with different amounts of P3HT as well as pure P3HT were identified by FTIR spectra (Figure 2a). The characteristic bands of pure P3HT (black curve) include 1,509 cm^−1^, 1,456 cm^−1^ (aromatic C=C stretching), 1,383 cm^−1^ (methyl bending), 1,118 cm^−1^ (C-S stretching), 821.6 cm^−1^ (aromatic C-H out-of-plane), and 722 cm^−1^ (methyl rock) [30]. For the CdSe sample prepared without P3HT ligands, the bands at approximately 1,119.2 and 1,383 cm^−1^ should be assigned to the stretching vibrations of C-S bond in DMSO and methyl in TCB from the solvent mixture, respectively. Interestingly, as the P3HT amount increases from 0 to 100 mg in the precursor solution, the band corresponding to C-S stretching vibration from the resulting CdSe sample shifts from 1,119.2 to 1,114 cm^−1^. This shift can be attributed to the light distortions of electronic cloud of the C-S bond away from the backbone of the P3HT chain, which resulted from the strong interaction between Cd^2+^ ions and S atoms that promotes the formation of coordination bond (Cd-S) and reduces C-S bond energy. A similar observation has been previously reported [30]. Based on the above results, it is concluded that there are P3HT ligands on the surface of CdSe superstructures prepared with the presence of 10 to 100 mg P3HT.

To evaluate the P3HT ligand content in CdSe superstructures prepared with different amounts of P3HT, TGA was performed (Figure 2b). For comparison, the TGA curve of pure P3HT (Figure 2b, black curve) was also recorded, and it shows that an initial decomposition occurs at 450°C and a sharp drop of the pure P3HT in weight percentage takes place at 500°C. Similarly, all these CdSe superstructures show weight loss between 450°C and 500°C, and the weight loss in this stage reflects the content of P3HT ligands. Obviously, with the increase of P3HT amount from 10 to 50 mg and then to 100 mg in the precursor solution, between 450°C and 500°C, the resulting CdSe superstructures exhibit the weight losses which go up from 0.5 to 10 wt.% and then to 12 wt.% of the total weight. These results indicate that the higher content of P3HT in the precursor solution results in more P3HT ligands in CdSe superstructures.

**Figure 3:**
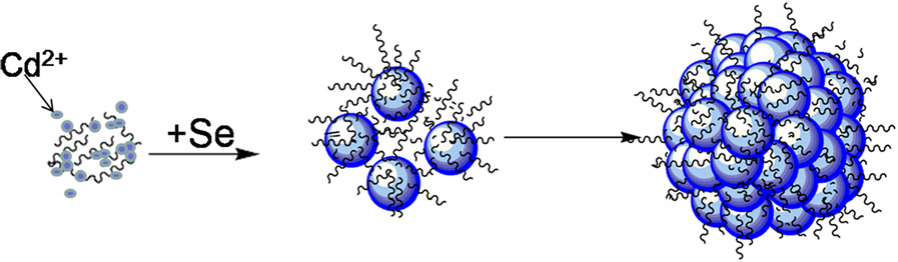
A proposed formation process for P3HT ligands on CdSe superstructures.

The formation mechanism of P3HT ligands on the surface of CdSe superstructures is proposed as follows (Figure 3). P3HT ligands have no obvious effect on shapes and phases of CdSe superstructures since the S atoms in the P3HT molecular chain have relatively mild coordination abilities with metal ions. When P3HT was dissolved in the solution containing Cd(CH_3_COO)_2_·2H_2_O, the S atoms of P3HT molecular chain and Cd^2+^ ions could form weak coordination bonds. After TCB solution containing Se powders was added, Cd^2+^ ions reacted with Se to produce CdSe nanoparticles. In the course of the reaction, P3HT molecules were coated onto the surfaces, resulting in an *in situ* generation of CdSe nanoparticles with the interaction between Cd^2+^ ions and the S atoms of the P3HT molecular chain. It has been reported that, although the formation of smaller crystallites was kinetically favored during the initial agglomeration, larger crystallites were thermodynamically favored [40]. Thus, during solvothermal treatment, the CdSe nanoparticles self-aggregated into the CdSe superstructure architectures (Figures 1c and 3). As a result of the presence of P3HT ligands on their surfaces, CdSe superstructures should have different optical properties compared with the samples without P3HT ligands.

**Figure 4:**
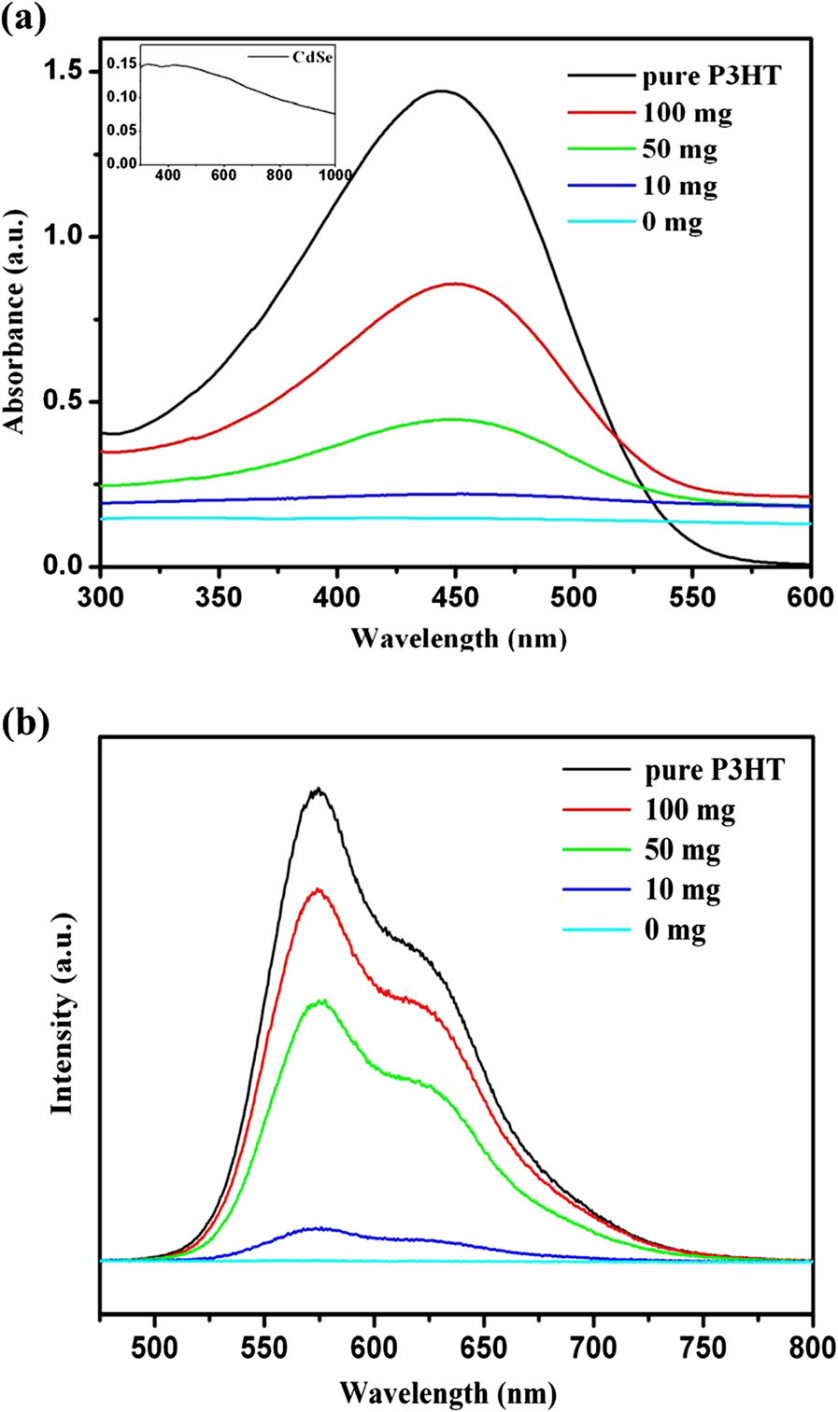
**UV–vis absorption spectra and PL spectra. (a)** The UV–vis absorption spectra (inset is the UV–vis absorption spectrum of CdSe and also the enlargement of light blue line) and **(b)** the PL spectra of the P3HT and the P3HT-capped CdSe superstructures synthesized with different amounts of P3HT at 0, 10, 50, and 100 mg.

Herein, we investigated the effects of the P3HT amount (0, 10, 50, and 100 mg) in the precursor solution on the photoabsorption and photoluminescence (PL) spectra of CdSe superstructures. Figure 4a presents the absorption spectra of the CHCl_3_ solution (0.04 mg/mL) containing CdSe superstructures, P3HT-capped CdSe superstructures, and pure P3HT. In the absence of P3HT ligands, CdSe superstructures exhibit weak absorption bands due to low concentration and weak absorption coefficient, as demonstrated in the light blue line in Figure 4a and the inset of Figure 4a. With the increase of the P3HT amount in the precursor solution from 10 to 100 mg, the absorption peak at about 445 nm goes up obviously, originating from the increased content and strong absorption coefficient of P3HT ligands. The corresponding PL spectra of these samples are measured at room temperature under the irradiation of 450-nm light (Figure 4b). The P3HT solution (black curve) exhibits a strong emission peak at 574 nm and a weaker emission peak at 624 nm. In the absence of P3HT ligands, CdSe superstructures exhibit weak emission bands due to low concentration and large-sized structures. With the increase of the P3HT amount from 10 to 100 mg in the precursor solution, the resulted CdSe superstructures exhibit significantly intensive emission peaks at 574 and 624 nm that are attributed to the emission of P3HT ligands. Thus, it can be concluded that the amount of P3HT in the precursor solution has a strong effect on the photoabsorption spectra and PL spectra, and a higher content of P3HT ligands in CdSe superstructures results in a stronger photoabsorption and PL emission intensity.

It is well known that traditional P3HT-CdSe hybrid solar cells have been constructed based on CdSe nanomaterials capped with organic aliphatic ligands, such as TOPO [24] and OA [16], and these aliphatic ligands prevent electron transferring from the photoexcited polymer to nanomaterials [25]. In our case, P3HT was used directly as the ligands of CdSe superstructures, and thus, the adverse effects of the capping ligands on charge exchange can be eliminated. In addition, CdSe superstructures constructed from CdSe nanoparticles with a diameter of 5 to 10 nm may be easy to form a well continuous inorganic network in a bulk heterojunction structure, probably resulting in the efficient electron transfer in inorganic network and the high photoelectric conversion efficiency.

**Figure 5:**
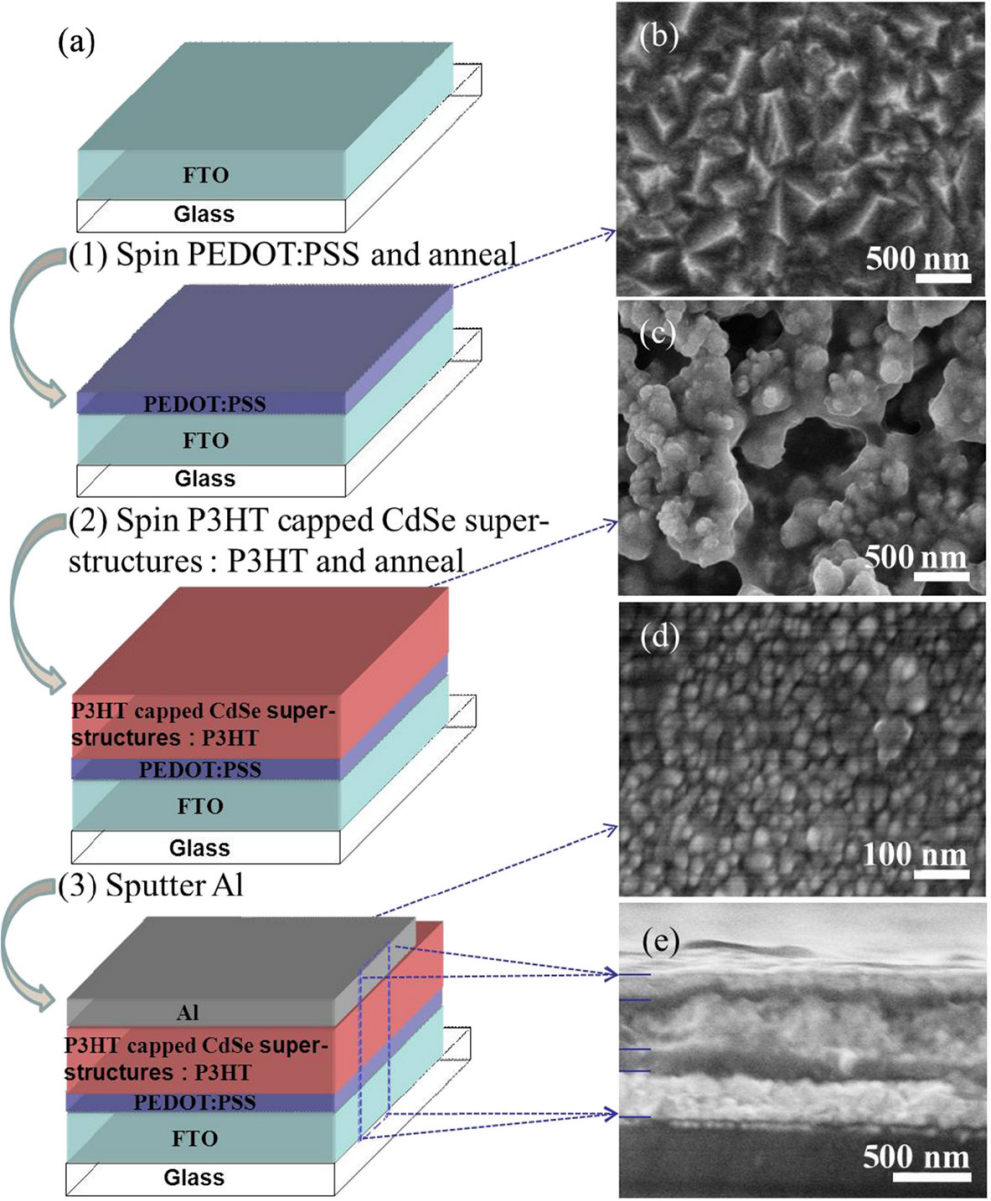
**Schematic illustration of solar cell fabrication and SEM images of solar cell. (a)** Schematic illustration of the fabrication of solar cell based on the P3HT-capped CdSe superstructures. SEM images **(b)** PEDOT:PSS film, **(c)** P3HT-capped CdSe superstructures and P3HT film, **(d)** Al film, **(e)** the cross-sectional view of the solar cell based on P3HT-capped CdSe superstructures synthesized with 50 mg P3HT.

**Figure 6:**
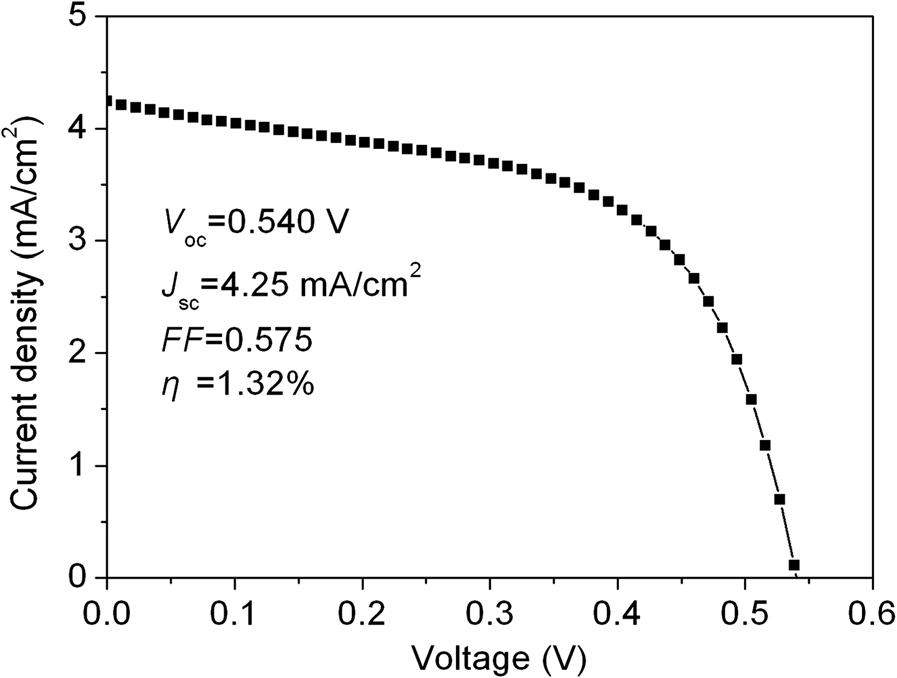
Photocurrent density-voltage characteristic of the solar cells fabricated by P3HT-capped CdSe superstructures.

Subsequently, P3HT-capped CdSe superstructures prepared in the presence of 50 mg P3HT were used as a model material to fabricate the solar cells with a structure of PEDOT:PSS/P3HT-capped CdSe superstructures:P3HT/Al. In a typical fabrication process (Figure 5a), the PEDOT:PSS layer (after annealing, Figure 5b) with a thickness of approximately 120 nm was prepared on FTO glass, and its surface was very rough, which is helpful for the adherence of absorption materials. CHCl_3_ solution containing P3HT (5 mg/mL) and P3HT-capped CdSe superstructures (20 mg/mL) was then used to fabricate the photoactive layer. This photoactive layer is compact and looks like a well continuous network (after annealing, Figure 5c). Finally, an Al layer with a thickness of 100 nm was sputtered as the cathode in the as-fabricated solar cell device (Figure 5d). The cross-sectional SEM image (Figure 5e) of the resulting cell exhibits a five-layer geometry, with a structure of glass/FTO/PEDOT:PSS (approximately 120 nm)/P3HT-capped CdSe superstructures: P3HT (approximately 450 nm)/Al (approximately 100 nm). Photocurrent density-voltage characteristics of the resulting solar cells based on CdSe superstructures with P3HT ligands are shown in Figure 6. The cell exhibited an open-circuit voltage (*V*_oc_) of 540 mV, a short-circuit current density (*J*_sc_) of 4.25 mA/cm^2^, and a fill factor (FF) of 57.5%, yielding an overall energy conversion efficiency (*η*) of 1.32%. This efficiency (approximately 1.3%) is not so high because of the holes/cracks formed within the films and uneven thickness of the films. Further improvement of the efficiency is ongoing by the optimization of the morphology and thickness of the films and the morphology of the P3HT and CdSe phases, as well as the fabrication technique of the device.

## Conclusions

In summary, an *in situ* growth method has been developed to synthesize P3HT-capped CdSe superstructures for their applications in solar cells. The amount of P3HT in the reaction solution has no obvious effect on the shapes and phases of CdSe superstructure samples, but the P3HT ligands in the CdSe superstructures promote the photoabsorption and PL emission intensities. The solar cell based on the P3HT-capped CdSe superstructures demonstrates an overall energy conversion efficiency (*η*) of 1.32%.

## Competing interests

The authors declare that they have no competing interests.

## Authors’ contributions

YP and GS carried out the laboratory experiments. XH and GH participated in the discussion of the results, analyzed the data, and drafted the manuscript. YP, JH, ZC, and XX conceived the study and participated in its design and coordination. All authors read and approved the final manuscript.
